# Erratum: *Lkb1* inactivation drives lung cancer lineage switching governed by Polycomb Repressive Complex 2

**DOI:** 10.1038/ncomms15901

**Published:** 2017-06-09

**Authors:** Haikuo Zhang, Christine Fillmore Brainson, Shohei Koyama, Amanda J. Redig, Ting Chen, Shuai Li, Manav Gupta, Carolina Garcia-de-Alba, Margherita Paschini, Grit S. Herter-Sprie, Gang Lu, Xin Zhang, Bryan P Marsh, Stephanie J. Tuminello, Chunxiao Xu, Zhao Chen, Xiaoen Wang, Esra A. Akbay, Mei Zheng, Sangeetha Palakurthi, Lynette M. Sholl, Anil K. Rustgi, David J. Kwiatkowski, J. Alan Diehl, Adam J. Bass, Norman E. Sharpless, Glenn Dranoff, Peter S. Hammerman, Hongbin Ji, Nabeel Bardeesy, Dieter Saur, Hideo Watanabe, Carla F. Kim, Kwok-Kin Wong

Nature Communications 8: Article number: 14922; DOI: 10.1038/ncomms14922 (2017); Published 04 07 2017; 06 09 2017

The affiliation details for Hideo Watanabe are incorrect in this Article. The correct affiliation details for this author are given below:

Department of Medicine, Division of Pulmonary, Critical Care and Sleep Medicine; Tisch Cancer Institute, Icahn School of Medicine at Mount Sinai, New York, New York 10029, USA.

