# Supplementary material for: Lkb1 inactivation drives lung cancer lineage switching governed by Polycomb Repressive Complex 2
Source: Nat Commun. 2017 Apr 7;8:14922. doi: 10.1038/ncomms14922 (PMC5385585; doi:10.1038/ncomms14922)
Supplement: Supplementary Information — Supplementary Figures and Supplementary Tables [file ncomms14922-s1.pdf]

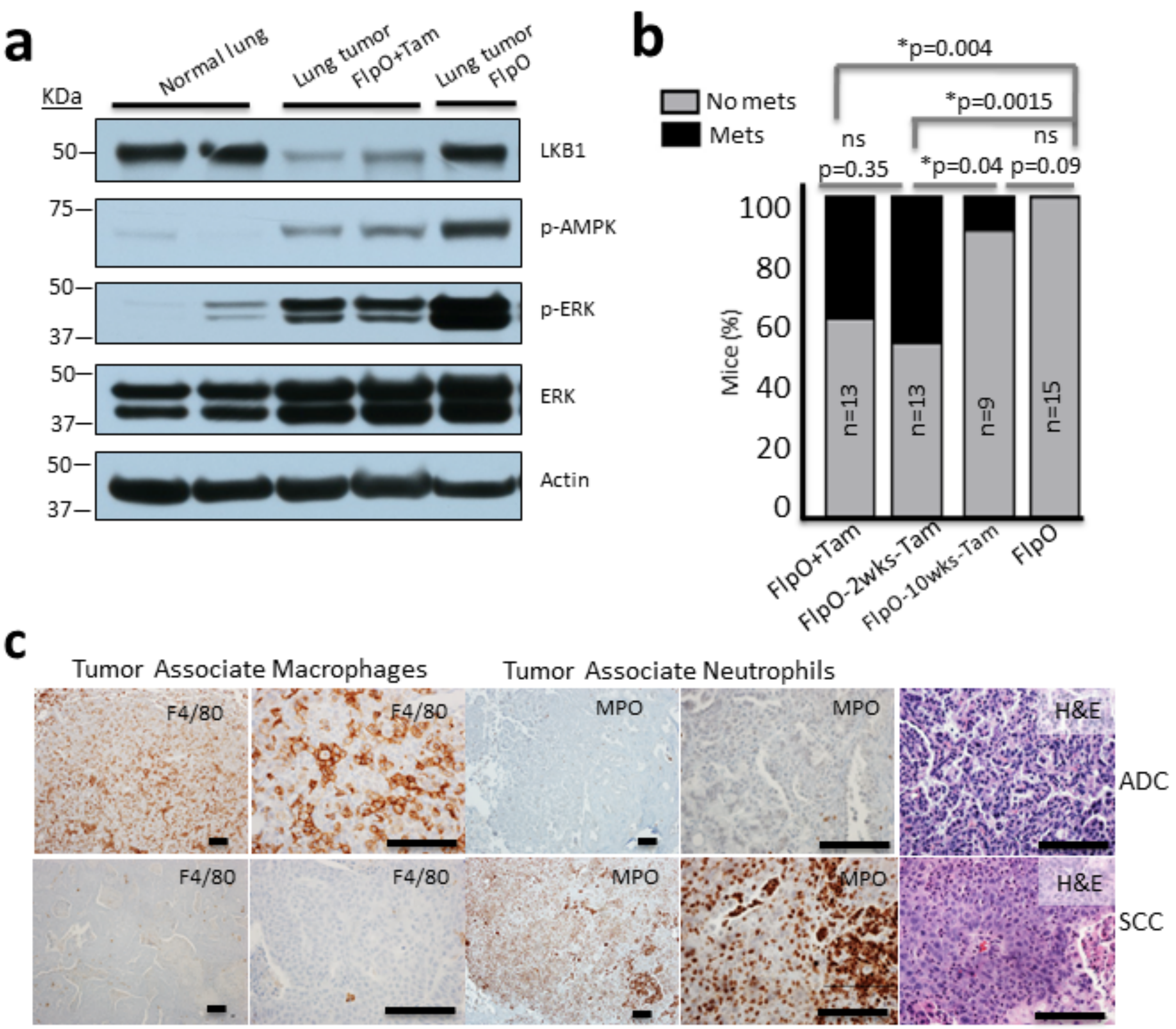

Supplementary Figure 1: *Lkb1* deletion drives SCC transition and tumor progression in established KRAS tumors

a) Western Blotting for LKB1, p-AMPK, p-ERK and total ERK in the indicated whole cell extracts. *Lkb1* deletion markedly decreases p-AMPK as expected. β-actin is the loading control. **b)** Incidence of metastasis within the cohorts as assessed by histologic examination of lymph nodes at endpoint, n and p values indicated on figure, p values represent Chi-squared test. **c)**

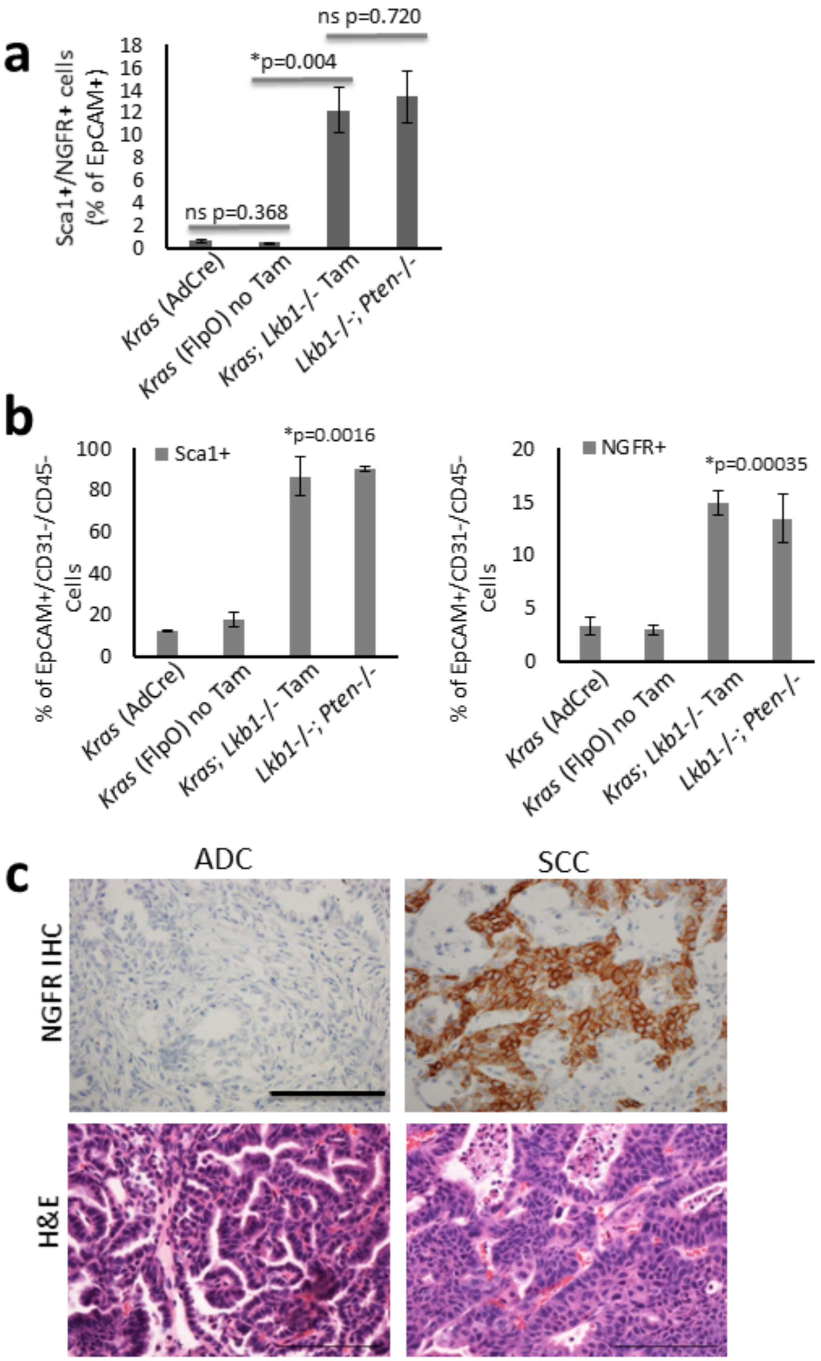

Supplementary Figure 2: *Lkb1* deletion after transplant of KRAS adenocarcinoma cells drives SCC transition

a) Flow cytometric analysis for average percentage of $NGFR^+/Sca1^+$ cells within the $DAPI^-/CD31^-/CD45^-/EpCAM^+$ of freshly dissociated tumors. Mean ± s.e.m. graphed, n=4 AdCre KRAS, n=3 no Tam, n=4 Tam, n=3 *Lkb1/Pten*, p values indicated on figure. **b)** Flow cytometric analysis for Sca1 and NGFR expression on the $DAPI^-/CD45^-/CD31^-/EpCAM^+$ cells of dissociated tumors from the indicated cohorts, mean ± s.e.m. graphed, n=4 AdCre KRAS, n=3 no Tam, n=4 Tam, n=3 *Lkb1/Pten*, p values for no Tam vs Tam comparison indicated on figure. **c)** Serial sections of hematoxylin and eosin staining of tumors paired with immunohistochemistry for NGFR, marking some epithelial cells in the post tamoxifen tumors, scale bar = 50μm.

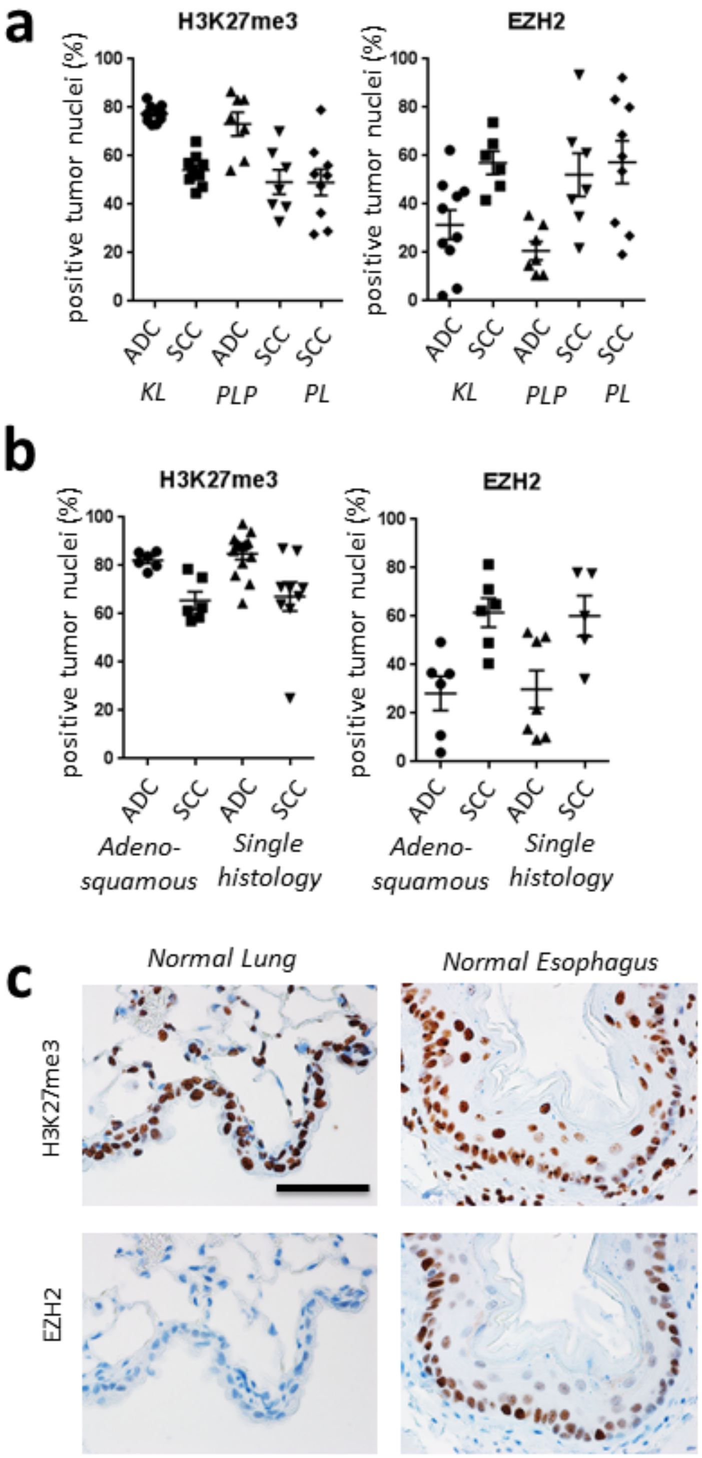

Supplementary Figure 3: Loss of histone H3 lysine 27 trimethylation accompanies SCC transition

a+b) More detailed graphical representation of the immunohistochemistry quantifications for EZH2 and H3K27me3 expression in the indicated tumor sections, **a)** murine tumors, **b)** human tumors. Mean ± s.e.m. graphed and each n can be observed on the graphs. **c)** Serial sections of immunohistochemistry for EZH2 and H3K27me3 in normal murine distal lung and esophagus, scale bar = 50µm.

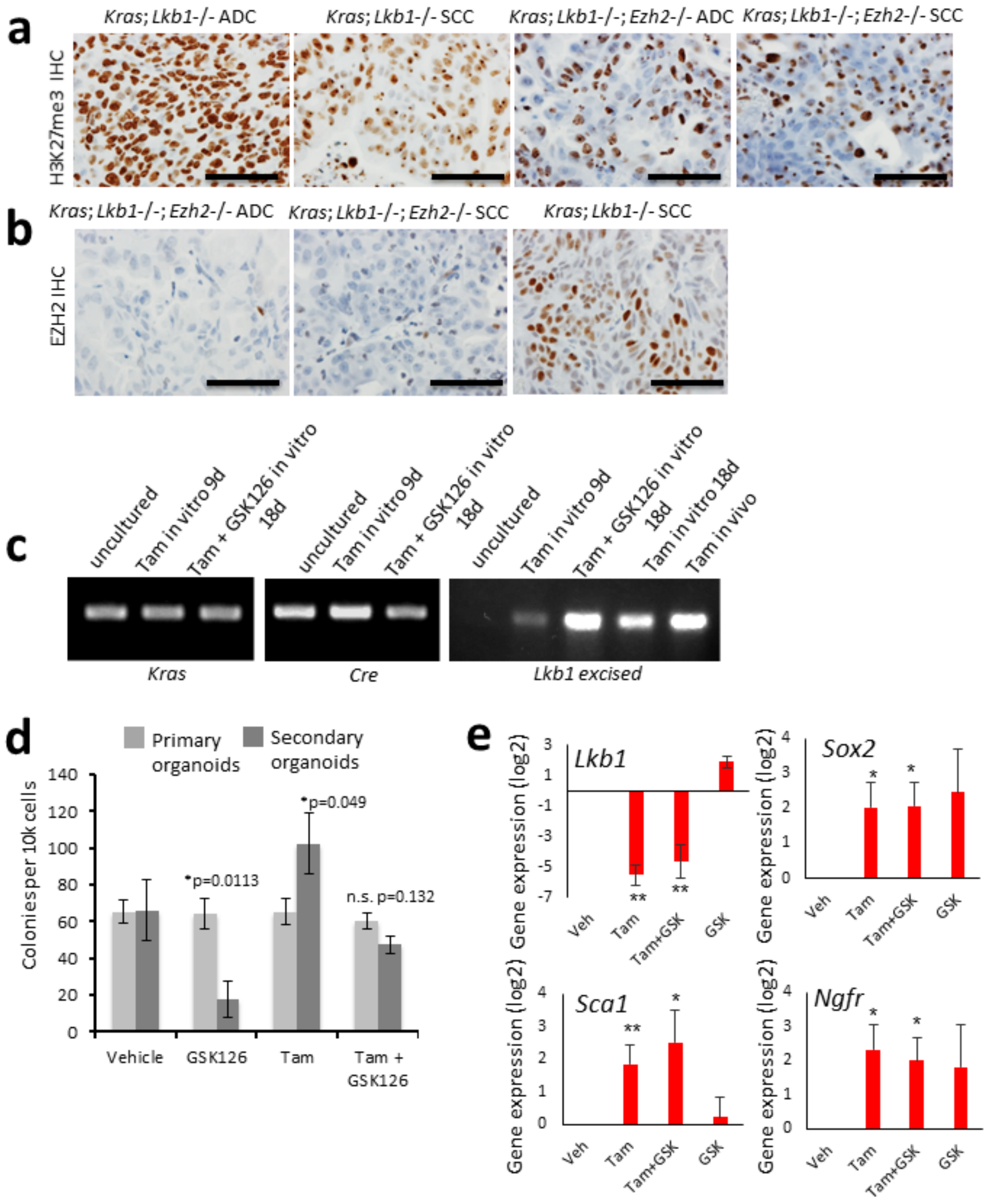

Supplementary Figure 4: Deletion or inhibition of EZH2 potentiates SCC transition

a) Immunohistochemistry for H3K27me3 in tumors of the indicated genotypes, scale bar = 50μm.

b) Immunohistochemistry for EZH2 in tumors of the indicated genotypes, scale bar = 50μm. **c)** PCR for alleles from the 3D tumor organoid cultures with the indicated treatments, *Kras* and *Cre* shown as controls, the excised *Lkb1* band is only present with tamoxifen treatment. **d)** Average number of colonies per 10,000 cells plated in the 3D cultures in the indicated treatments, mean ± s.e.m. graphed, n=5 for primary cultures, n=3 for secondary cultures, p values indicated on figure. **e)** RT-qPCR for *Sox2*, *Sca1* and *Lkb1* in secondary tumor organoid 3D cultures treated with 100nM tamoxifen, 5μM of EZH2 inhibitor GSK126, or both for 18-24 days total, mean ± s.e.m. on log_2 scale is graphed, ** indicates p<0.01, * indicates p<0.05, n=4 except for GSK, n=2.

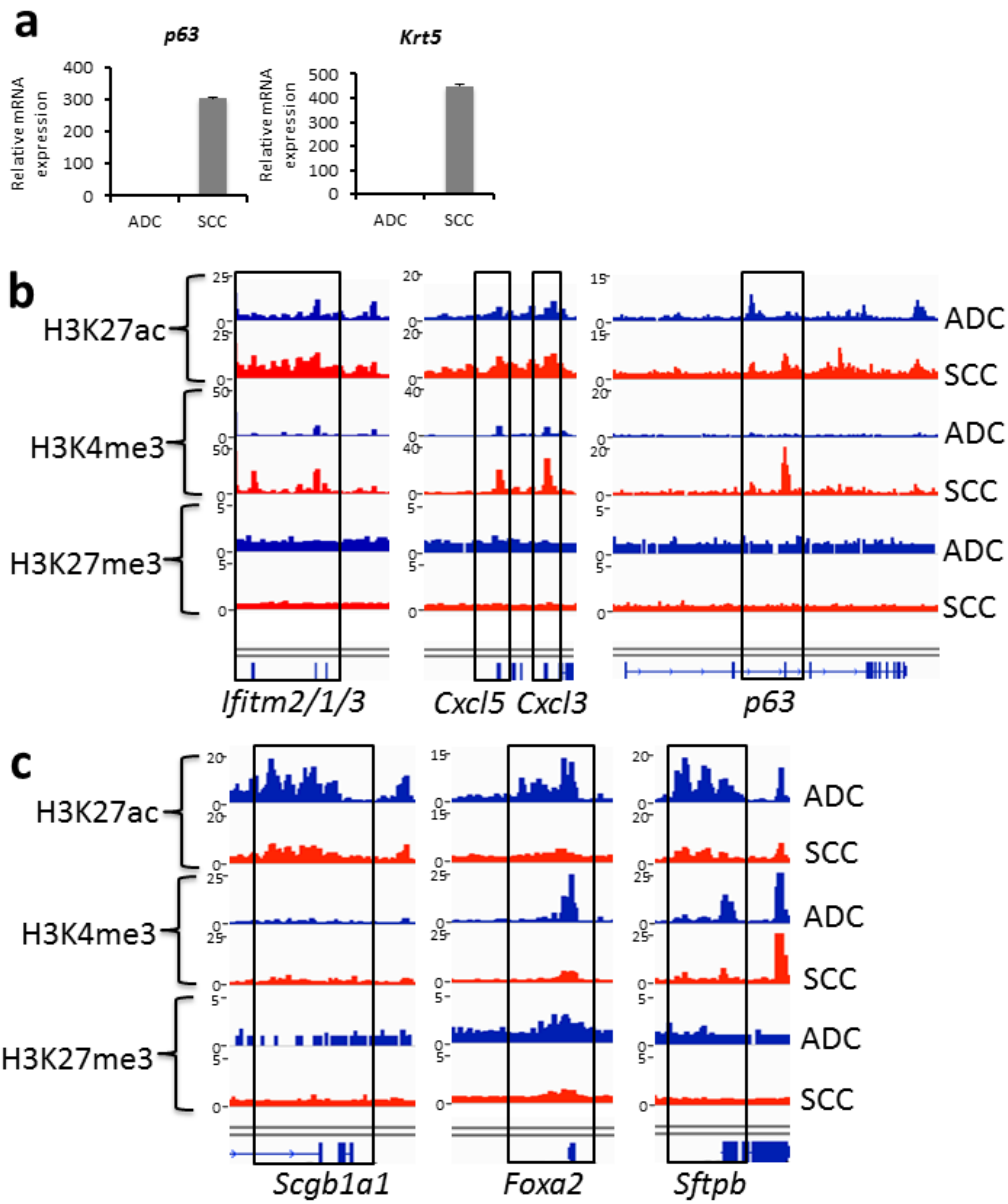

Supplementary Figure 5: Chromatin landscapes of ADC and SCCs reveal de-repression of squamous genes

a) RT-qPCR for expression the squamous genes *p63* and *Krt5* to confirm subtypes of tumors used for ChIP experiments. **b)** Genome browser snapshots of the squamous loci *Ifitm3, Cxcl3/5 and p63* with the indicated ChIP-sequencing peaks for H3K27ac, H3K27me3 and H3K4me3 in the ADC (blue) and SCC (red) KRAS*/Lkb1* tumor samples. **c)** Genome browser snapshots of the squamous loci *Scgb1a1*, *Foxa2* and *Sftpb* with the indicated ChIP-sequencing peaks for H3K27ac, H3K27me3 and H3K4me3 in the ADC (blue) and SCC (red) KRAS*/Lkb1* tumor samples.

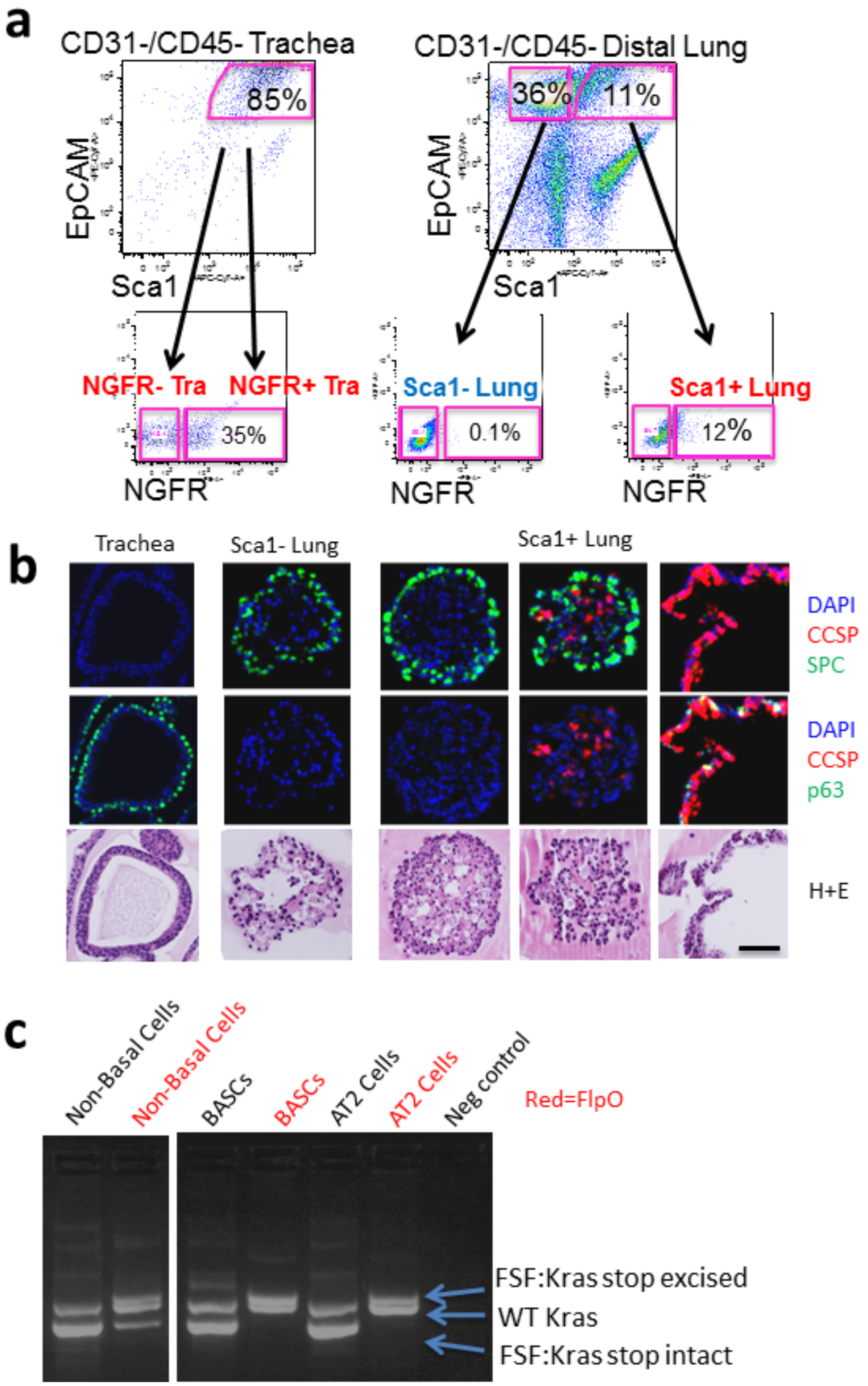

Supplementary Figure 6: KRAS activation is tolerated by Club Cell, BASC and AT2 Cell populations

a) Flow cytometry scheme for isolating stem and progenitor populations. Basal cells are isolated from tracheal cell preparations by exclusion of CD31 and CD45, and gating on EpCAM+, Sca1+ and NGFR+ cells. Non-basal cells are EpCAM+ and Sca1+ but NGFR-. In the distal lung preparations, again CD31 and CD45 are excluded, prior to gating on EpCAM+ cells. Sca1- cells are enriched for AT2 cells, which are uniformly NGFR- in the normal mouse. BASCs are Sca1+ and some express NGFR. We used both NGFR+ and NGFR- BASCs for our experiments. **b)** Representative immunofluoresence for the cell lineage markers CCSP, SPC and p63 in indicated organoid cultures sections with hematoxylin and eosin staining below, scale bar = 50μm. **c)** PCR for *Kras* excision in organoid cultures derived from the indicated sorted lung cell populations.

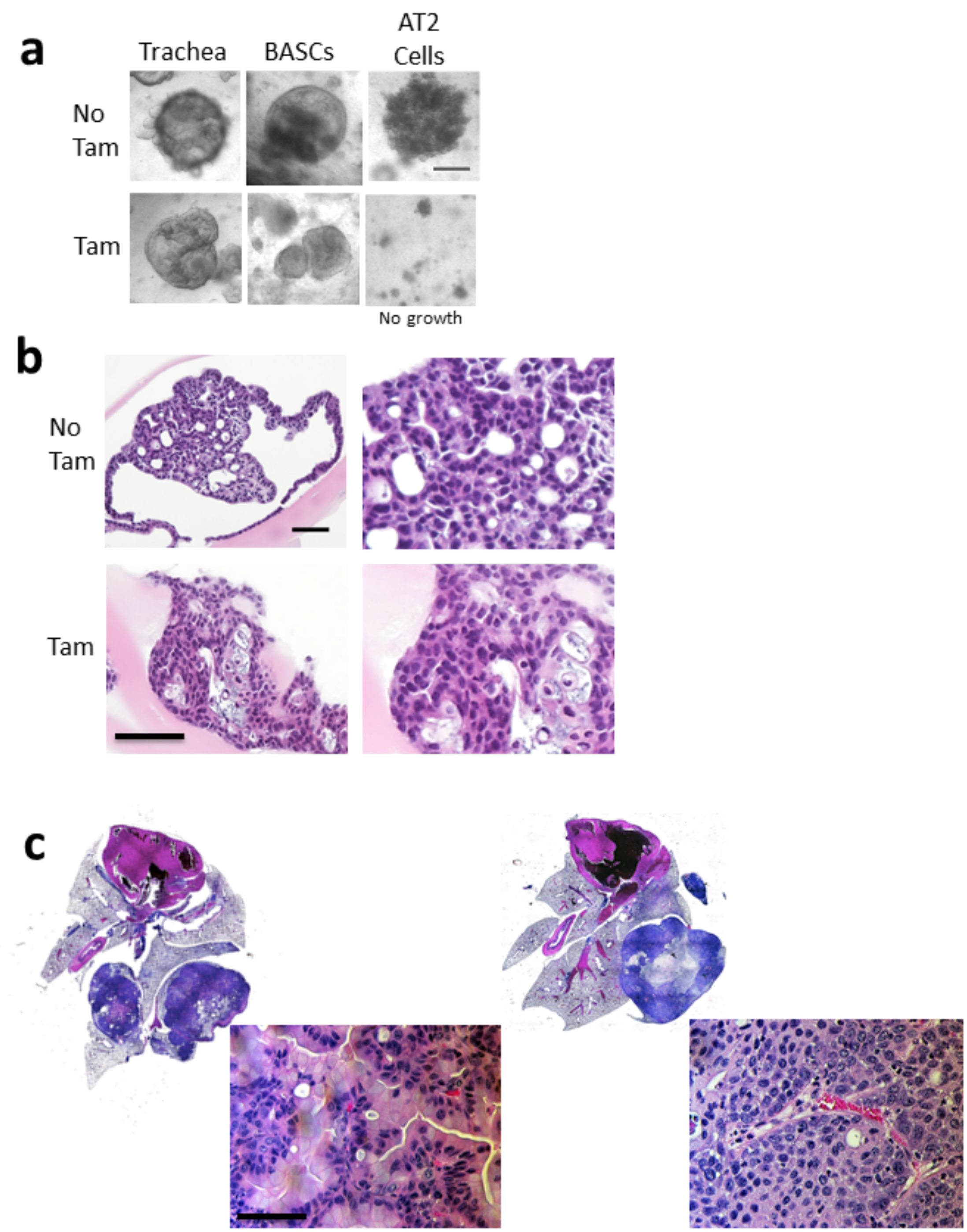

Supplementary Figure 7: *Lkb1* Deletion Drives Squamous Transition of Club Cell- and BASC-derived KRAS+ populations

a) Representative brightfield images of KRAS+ colonies from the indicated cell types, scale bar = 100µm. **b)** Representative hematoxylin and eosin staining of sections from tracheal derived organoids treated with the indicated tamoxifen or not for 9 days, scale bar = 100µm. **c)** Whole lung histology, with representative images of magnified histology of the tumor from each lung, from BASC derived orthotopically transplanted KRAS/*Lkb1* cells, scale bar = 50µm.

Supplementary Table 1: *Unique H3K27ac peaks in SCC samples*

Unique somatic peaks associated with protein coding genes, top 185 of a possible 246 with Log2FC>2.5, p value<1E-10

LOG2FC	Negative LOG10 (p value)	Gene Name	Gene Description
6.259	300.000	Tmem45b	transmembrane protein 45b
5.987	300.000	Mroh5	maestro heat-like repeat family member 5
5.985	275.755	Pitx1	paired-like homeodomain transcription factor 1
5.704	71.065	Pitx1	paired-like homeodomain transcription factor 1
5.496	25.304	Chst9	carbohydrate (N-acetylgalactosamine 4-0) sulfotransferase 9
5.496	25.304	Fut9	fucosyltransferase 9
5.183	29.366	Foxd4	forkhead box D4
5.119	27.901	Dgkg	diacylglycerol kinase, gamma
5.099	44.700	Slitrk6	SLIT and NTRK-like family, member 6
5.086	27.316	Tmprss11g	transmembrane protease, serine 11g
5.018	25.854	Barx2	BarH-like homeobox 2
4.915	38.893	Chst9	carbohydrate (N-acetylgalactosamine 4-0) sulfotransferase 9
4.841	36.578	Fabp12	fatty acid binding protein 12
4.716	20.612	Chst9	carbohydrate (N-acetylgalactosamine 4-0) sulfotransferase 9
4.683	32.252	Cpne8	copine VIII
4.671	25.993	Ccdc91	coiled-coil domain containing 91
4.603	24.552	Car12	carbonic anyhydrase 12
4.594	104.904	Engase	endo-beta-N-acetylglucosaminidase
4.588	18.584	Sox2	SRY (sex determining region Y)-box 2
4.415	26.237	Gpr115	G protein-coupled receptor 115
4.381	20.538	A1bg	alpha-1-B glycoprotein
4.335	29.096	Tnc	tenascin C
4.304	32.817	Lbx1	ladybird homeobox homolog 1 (Drosophila)
4.273	40.836	Pyroxd1	pyridine nucleotide-disulphide oxidoreductase domain 1
4.262	49.142	Ndfip2	Nedd4 family interacting protein 2
4.210	22.261	Hlf	hepatic leukemia factor
4.179	78.192	Pitx1	paired-like homeodomain transcription factor 1
4.174	21.414	Pitx1	paired-like homeodomain transcription factor 1
4.098	20.287	Tmprss11a	transmembrane protease, serine 11a
4.074	89.946	Ccdc91	coiled-coil domain containing 91
4.053	95.768	S100a9	S100 calcium binding protein A9 (calgranulin B)
4.035	15.713	Mfsd10	major facilitator superfamily domain containing 10
4.023	26.079	Cpne8	copine VIII
3.890	26.490	Tmprss11g	transmembrane protease, serine 11g
3.800	24.287	Pthlh	parathyroid hormone-like peptide
3.796	18.456	Irf5	interferon regulatory factor 5
3.796	18.456	Tmprss11g	transmembrane protease, serine 11g
3.796	21.371	Secisbp2	SECIS binding protein 2
3.796	21.371	Sord	sorbitol dehydrogenase
3.713	14.714	Marcks	myristoylated alanine rich protein kinase C substrate
3.703	43.557	Ehf	ets homologous factor
3.699	38.259	Six1	sine oculis-related homeobox 1
3.686	58.643	Phospho1	phosphatase, orphan 1
3.679	61.023	S100a9	S100 calcium binding protein A9 (calgranulin B)
3.658	82.717	Helt	helt bHLH transcription factor
3.652	28.958	Prl2c5	prolactin family 2, subfamily c, member 5
3.635	85.867	S100a9	S100 calcium binding protein A9 (calgranulin B)
3.588	39.244	Cdh6	cadherin 6
3.564	12.796	Sc5d	sterol-C5-desaturase (fungal ERG3, delta-5-desaturase) homolog (S. cerevisae)
3.517	25.727	Pthlh	parathyroid hormone-like peptide
3.503	49.531	S100a8	S100 calcium binding protein A8 (calgranulin A)
3.501	32.071	Pyroxd1	pyridine nucleotide-disulphide oxidoreductase domain 1
3.501	27.573	Trp63	transformation related protein 63
3.496	18.582	Zfand2b	zinc finger, AN1 type domain 2B
3.489	25.191	Rps23	ribosomal protein S23
3.452	15.932	Frmd6	FERM domain containing 6
3.450	13.820	Capns2	calpain, small subunit 2
3.437	44.498	Ehf	ets homologous factor
3.425	31.794	Ppp1r3b	protein phosphatase 1, regulatory (inhibitor) subunit 3B
3.418	41.852	Olfr52	olfactory receptor 52
3.398	37.089	S100a9	S100 calcium binding protein A9 (calgranulin B)
3.366	14.595	Rcn1	reticulocalbin 1
3.352	12.479	Amotl1	angiomotin-like 1
3.342	16.180	C9	complement component 9
3.342	16.180	Ccdc91	coiled-coil domain containing 91
3.340	21.739	Fbxl14	F-box and leucine-rich repeat protein 14
3.332	31.012	Gm5483	predicted gene 5483
3.326	17.768	Il1b	interleukin 1 beta

3.305	37.649	Gm5483	predicted gene 5483
3.290	31.559	Tnc	tenascin C
3.281	18.833	Qsox2	quiescin Q6 sulfhydryl oxidase 2
3.276	22.281	Cpeb2	cytoplasmic polyadenylation element binding protein 2
3.275	13.534	Rps23	ribosomal protein S23
3.259	15.124	Arhgap5	Rho GTPase activating protein 5
3.259	15.124	Gab1	growth factor receptor bound protein 2-associated protein 1
3.248	23.614	Pitx1	paired-like homeodomain transcription factor 1
3.248	23.614	Ranbp6	RAN binding protein 6
3.246	18.309	Ipcef1	interaction protein for cytohesin exchange factors 1
3.246	18.309	Rbbp6	retinoblastoma binding protein 6
3.217	14.336	Chac1	ChaC, cation transport regulator 1
3.217	14.336	Entpd3	ectonucleoside triphosphate diphosphohydrolase 3
3.217	14.336	Perp	PERP, TP53 apoptosis effector
3.203	47.308	Vsnl1	visinin-like 1
3.200	175.564	Csf3r	colony stimulating factor 3 receptor (granulocyte)
3.197	23.911	Il1b	interleukin 1 beta
3.191	40.144	Il1r2	interleukin 1 receptor, type II
3.189	53.185	Supt20	suppressor of Ty 20
3.174	17.005	Irf6	interferon regulatory factor 6
3.166	263.729	Csf3r	colony stimulating factor 3 receptor (granulocyte)
3.165	48.704	Cpeb2	cytoplasmic polyadenylation element binding protein 2
3.164	32.722	Trp63	transformation related protein 63
3.160	21.537	Oasl2	2'-5' oligoadenylate synthetase-like 2
3.160	21.537	Pthlh	parathyroid hormone-like peptide
3.119	19.166	Irg1	immunoresponsive gene 1
3.099	20.250	Bcl2l15	BCLl2-like 15
3.093	32.016	Fam122a	family with sequence similarity 122, member A
3.088	33.359	Tas2r126	taste receptor, type 2, member 126
3.069	60.172	Cdca7	cell division cycle associated 7
3.060	15.199	Gzmk	granzyme K
3.060	15.199	Slamf6	SLAM family member 6
3.059	23.768	Oacyl	O-acyltransferase like
3.055	29.399	Slco1a6	solute carrier organic anion transporter family, member 1a6
3.053	74.967	Upp1	uridine phosphorylase 1
3.052	18.140	Rps27a	ribosomal protein S27A
3.036	16.286	Tmppe	transmembrane protein with metallophosphoesterase domain
3.032	23.260	Tbc1d8	TBC1 domain family, member 8
3.021	51.760	S100a9	S100 calcium binding protein A9 (calgranulin B)
3.019	50.415	Ptpn22	protein tyrosine phosphatase, non-receptor type 22 (lymphoid)
3.018	25.695	Il1b	interleukin 1 beta
3.018	17.375	1810011O10Rik	RIKEN cDNA 1810011O10 gene
3.013	59.582	S100a8	S100 calcium binding protein A8 (calgranulin A)
3.011	96.166	Ccdc91	coiled-coil domain containing 91
2.983	32.758	Zfyve26	zinc finger, FYVE domain containing 26
2.972	23.088	Krt5	keratin 5
2.970	17.959	Dgkh	diacylglycerol kinase, eta
2.963	26.873	Iyd	iodotyrosine deiodinase
2.962	28.219	Ccpg1	cell cycle progression 1
2.961	15.269	Syt6	synaptotagmin VI
2.952	68.786	Csf3r	colony stimulating factor 3 receptor (granulocyte)
2.948	35.292	Mmp25	matrix metallopeptidase 25
2.940	25.026	Marcks	myristoylated alanine rich protein kinase C substrate
2.932	74.870	Klhl6	kelch-like 6
2.923	89.032	Tas2r143	taste receptor, type 2, member 143
2.902	16.701	Grhl1	grainyhead-like 1 (Drosophila)
2.897	17.797	Tbcd	tubulin-specific chaperone d
2.893	18.893	Meis2	Meis homeobox 2
2.890	20.238	Cxcl2	chemokine (C-X-C motif) ligand 2
2.890	75.192	Il1r2	interleukin 1 receptor, type II
2.880	113.658	Bcl2l15	BCLl2-like 15
2.876	171.180	Zfp715	zinc finger protein 715
2.868	31.460	Musk	muscle, skeletal, receptor tyrosine kinase
2.864	34.754	Cpeb2	cytoplasmic polyadenylation element binding protein 2
2.861	18.395	Ehf	ets homologous factor
2.861	18.395	Gm17455	predicted gene, 17455
2.845	24.981	Eea1	early endosome antigen 1
2.843	92.263	Il1b	interleukin 1 beta
2.842	13.510	Jazf1	JAZF zinc finger 1
2.836	28.029	Pvrl1	poliovirus receptor-related 1
2.835	14.606	Gxylt1	glucoside xylosyltransferase 1
2.824	144.041	Ifitm3	interferon induced transmembrane protein 3
2.824	45.610	Kdm2a	lysine (K)-specific demethylase 2A
2.814	27.538	Oasl2	2'-5' oligoadenylate synthetase-like 2
2.786	34.251	Pdzd2	PDZ domain containing 2
2.785	38.404	Rassf6	Ras association (RalGDS/AF-6) domain family member 6
2.784	21.307	Cdca7	cell division cycle associated 7
2.778	24.359	Marcks	myristoylated alanine rich protein kinase C substrate

2.777	39.260	Trp63	transformation related protein 63
2.776	117.930	Arhgap5	Rho GTPase activating protein 5
2.764	16.666	Acot1	acyl-CoA thioesterase 1
2.761	30.223	Atf1	activating transcription factor 1
2.760	11.419	Serpinb10	serine (or cysteine) peptidase inhibitor, clade B (ovalbumin), member 10
2.757	20.574	Zfp715	zinc finger protein 715
2.749	32.792	Cxcl5	chemokine (C-X-C motif) ligand 5
2.745	22.528	Fam122a	family with sequence similarity 122, member A

Supplementary Table 2: *Unique H3K4me3 peaks in SCC samples*

Unique somatic peaks associated with protein coding genes, top 185 with an additional 1 selected locus, of a possible 584 with Log2FC>1.4, p value<1E-10

LOG2FC	Negative LOG10 (p value)	Gene Name	Gene Description
6.947	72.129	Tmprss11b	transmembrane protease, serine 11B
6.101	207.438	Frmd6	FERM domain containing 6
5.849	110.129	Ccdc91	coiled-coil domain containing 91
5.760	74.011	Cfap20	cilia and flagella associated protein 20
5.412	56.966	Tmprss11g	transmembrane protease, serine 11g
5.171	157.277	Krt17	keratin 17
5.108	53.837	Ppbp	pro-platelet basic protein
4.986	33.237	Dsc2	desmocollin 2
4.790	35.134	Ptprm	protein tyrosine phosphatase, receptor type, M
4.786	189.576	Slitrk6	SLIT and NTRK-like family, member 6
4.781	68.456	Foxd4	forkhead box D4
4.774	28.015	Jazf1	JAZF zinc finger 1
4.738	117.737	Dst	dystonin
4.542	89.523	Fat2	FAT tumor suppressor homolog 2 (Drosophila)
4.523	50.179	Trp73	transformation related protein 73
4.476	58.742	Tmprss11g	transmembrane protease, serine 11g
4.452	307.626	Trp63	transformation related protein 63
4.372	87.353	Arhgef16	Rho guanine nucleotide exchange factor (GEF) 16
4.371	39.651	Arhgap30	Rho GTPase activating protein 30
4.271	45.129	Pkp1	plakophilin 1
4.201	38.587	Pthlh	parathyroid hormone-like peptide
4.164	25.424	Rad51b	RAD51 homolog B
4.134	28.595	Slc35c1	solute carrier family 35, member C1
4.130	315.651	Krt5	keratin 5
4.112	69.998	Ccdc91	coiled-coil domain containing 91
4.098	141.072	Pthlh	parathyroid hormone-like peptide
3.974	21.504	Aqp4	aquaporin 4
3.880	47.999	Amotl1	angiomotin-like 1
3.842	34.410	Il1b	interleukin 1 beta
3.793	96.179	Pex10	peroxisomal biogenesis factor 10
3.786	253.246	Csf3r	colony stimulating factor 3 receptor (granulocyte)
3.769	187.113	Grik3	glutamate receptor, ionotropic, kainate 3
3.705	80.920	Spsb1	splA/ryanodine receptor domain and SOCS box containing 1
3.685	27.394	Tmprss11b	transmembrane protease, serine 11B
3.620	62.555	Adcyap1	adenylate cyclase activating polypeptide 1
3.615	47.730	Padi3	peptidyl arginine deiminase, type III
3.611	125.555	Mrgpra2a	MAS-related GPR, member A2A
3.605	30.257	Tmpo	thymopoietin
3.605	156.522	Irg1	immunoresponsive gene 1
3.596	78.156	D1Ertd622e	DNA segment, Chr 1, ERATO Doi 622, expressed
3.590	27.608	Sord	sorbitol dehydrogenase
3.564	43.203	Dcaf4	DDB1 and CUL4 associated factor 4
3.557	140.324	Chst11	carbohydrate sulfotransferase 11
3.547	33.408	Cdk5rap1	CDK5 regulatory subunit associated protein 1
3.525	52.978	S100a9	S100 calcium binding protein A9 (calgranulin B)
3.519	97.433	Clca5	chloride channel calcium activated 5
3.503	45.299	Krt6b	keratin 6B
3.494	125.745	Mrgpra2b	MAS-related GPR, member A2B
3.481	33.653	Bnc1	basonuclin 1
3.464	164.921	Dsg3	desmoglein 3
3.424	89.236	Cxcr2	chemokine (C-X-C motif) receptor 2
3.420	213.146	Spatc1	spermatogenesis and centriole associated 1
3.415	59.854	S100a7a	S100 calcium binding protein A7A
3.408	245.205	Nlrp12	NLR family, pyrin domain containing 12
3.402	63.300	S100a8	S100 calcium binding protein A8 (calgranulin A)
3.383	28.619	Pitx1	paired-like homeodomain transcription factor 1
3.378	24.649	Tcerg1	transcription elongation regulator 1 (CA150)
3.372	49.807	Galm	galactose mutarotase
3.352	224.445	Amer2	APC membrane recruitment 2
3.350	33.392	Prickle2	prickle homolog 2 (Drosophila)

3.346	122.696	Galnt6	UDP-N-acetyl-alpha-D-galactosamine:polypeptide N-acetylgalactosaminyltransferase 6
3.334	232.194	Krt85	keratin 85
3.334	32.868	Gm884	predicted gene 884
3.321	28.895	Nlrp12	NLR family, pyrin domain containing 12
3.320	95.441	Retnlg	resistin like gamma
3.301	30.226	Amer2	APC membrane recruitment 2
3.280	63.405	Serpinb10	serine (or cysteine) peptidase inhibitor, clade B (ovalbumin), member 10
3.268	53.866	Ltf	lactotransferrin
3.239	286.676	S100a8	S100 calcium binding protein A8 (calgranulin A)
3.235	87.925	Sik1	salt inducible kinase 1
3.235	87.925	Stfa2l1	stefin A2 like 1
3.235	55.506	Asb7	ankyrin repeat and SOCS box-containing 7
3.218	210.553	S100a9	S100 calcium binding protein A9 (calgranulin B)
3.218	22.833	Dusp6	dual specificity phosphatase 6
3.217	51.278	Pola2	polymerase (DNA directed), alpha 2
3.217	51.278	Rassf6	Ras association (RalGDS/AF-6) domain family member 6
3.208	112.205	Fabp5	fatty acid binding protein 5, epidermal
3.178	23.652	Prps1l1	phosphoribosyl pyrophosphate synthetase 1-like 1
3.169	44.166	Tmprss13	transmembrane protease, serine 13
3.140	30.610	Cxcr1	chemokine (C-X-C motif) receptor 1
3.138	19.682	Ptgdr	prostaglandin D receptor
3.137	41.286	Ltf	lactotransferrin
3.132	31.695	AF251705	cDNA sequence AF251705
3.113	49.344	Lin28a	lin-28 homolog A (C. elegans)
3.100	23.447	Sgms2	sphingomyelin synthase 2
3.093	72.120	Dmxl2	Dmx-like 2
3.092	42.440	Arfgef1	ADP-ribosylation factor guanine nucleotide-exchange factor 1(brefeldin A-inhibited)
3.091	24.534	Pvrl1	poliovirus receptor-related 1
3.090	37.900	Klhdc4	kelch domain containing 4
3.076	51.850	Pdzd2	PDZ domain containing 2
3.062	30.997	Fbln1	fibulin 1
3.053	28.055	Dmkn	dermokine
3.050	63.195	Asb7	ankyrin repeat and SOCS box-containing 7
3.040	33.179	Tmprss11e	transmembrane protease, serine 11e
3.040	26.202	Tnfrsf1a	tumor necrosis factor receptor superfamily, member 1a
3.026	115.669	Asprv1	aspartic peptidase, retroviral-like 1
3.001	96.509	Hcar2	hydroxycarboxylic acid receptor 2
2.985	48.656	Dapl1	death associated protein-like 1
2.983	57.826	Dgat2	diacylglycerol O-acyltransferase 2
2.980	123.616	Slfn4	schlafen 4
2.973	55.979	Trak1	trafficking protein, kinesin binding 1
2.953	36.888	Hist2h2aa2	histone cluster 2, H2aa2
2.949	32.850	Dmxl2	Dmx-like 2
2.943	177.304	Wfdc21	WAP four-disulfide core domain 21
2.941	19.894	Sfn	stratifin
2.932	147.702	Il1f9	interleukin 1 family, member 9
2.932	51.042	Gda	guanine deaminase
2.924	68.142	Hsd17b2	hydroxysteroid (17-beta) dehydrogenase 2
2.906	25.372	Ly6c2	lymphocyte antigen 6 complex, locus C2
2.904	46.012	Dennd4a	DENN/MADD domain containing 4A
2.892	18.893	Bmp2k	BMP2 inducible kinase
2.880	73.354	Styxl1	serine/threonine/tyrosine interacting-like 1
2.867	186.070	Il1b	interleukin 1 beta
2.836	16.801	Plekhs1	pleckstrin homology domain containing, family S member 1
2.832	101.420	Cd177	CD177 antigen
2.823	43.412	Pvrl1	poliovirus receptor-related 1
2.814	49.517	BC117090	cDNA sequence BC1179090
2.807	61.363	Eef1a1	eukaryotic translation elongation factor 1 alpha 1
2.790	22.406	Gadd45b	growth arrest and DNA-damage-inducible 45 beta
2.787	179.485	Trim30b	tripartite motif-containing 30B
2.780	117.316	Fam167a	family with sequence similarity 167, member A
2.779	31.809	BC100530	cDNA sequence BC100530
2.773	70.778	Tnfsf14	tumor necrosis factor (ligand) superfamily, member 14
2.770	28.268	Ppp1r42	protein phosphatase 1, regulatory subunit 42
2.769	99.002	Tarm1	T cell-interacting, activating receptor on myeloid cells 1
2.768	35.719	Ear10	eosinophil-associated, ribonuclease A family, member 10
2.759	48.795	Csf3r	colony stimulating factor 3 receptor (granulocyte)
2.759	48.795	Pcsk9	proprotein convertase subtilisin/kexin type 9
2.756	175.633	Eea1	early endosome antigen 1
2.754	235.515	C1qtnf9	C1q and tumor necrosis factor related protein 9
2.748	72.139	Cep19	centrosomal protein 19
2.738	20.330	Angpt2	angiopoietin 2
2.731	23.142	Psca	prostate stem cell antigen
2.728	89.644	Meis2	Meis homeobox 2
2.719	21.800	Coq7	demethyl-Q 7
2.718	80.613	Mmp8	matrix metallopeptidase 8
2.716	130.265	Chil1	chitinase-like 1
2.708	52.252	Marcks	myristoylated alanine rich protein kinase C substrate

2.699	57.643	Txnl4b	thioredoxin-like 4B
2.693	35.873	Stfa2	stefin A2
2.690	65.613	Gm5483	predicted gene 5483
2.679	27.564	Dusp6	dual specificity phosphatase 6
2.673	70.290	Syk	spleen tyrosine kinase
2.660	144.920	LOC100038947	signal-regulatory protein beta 1-like
2.654	41.789	Srgn	serglycin
2.650	57.355	Syne1	spectrin repeat containing, nuclear envelope 1
2.636	16.439	Tmpo	thymopoietin
2.635	39.117	Ofcc1	orofacial cleft 1 candidate 1
2.619	47.243	Rnf149	ring finger protein 149
2.616	33.868	Rnf157	ring finger protein 157
2.615	19.636	Trem1	triggering receptor expressed on myeloid cells 1
2.606	143.525	Pitx1	paired-like homeodomain transcription factor 1
2.604	87.911	Arhgap32	Rho GTPase activating protein 32
2.599	234.960	Gda	guanine deaminase
2.595	26.901	Pgbd5	piggyBac transposable element derived 5
2.593	19.160	Pde6h	phosphodiesterase 6H, cGMP-specific, cone, gamma
2.588	20.643	B430306N03Rik	RIKEN cDNA B430306N03 gene
2.584	22.363	Atl2	atlastin GTPase 2
2.577	101.440	Vsnl1	visinin-like 1
2.566	209.788	Adam8	a disintegrin and metallopeptidase domain 8
2.564	23.612	Zfp715	zinc finger protein 715
2.560	208.154	Clec4e	C-type lectin domain family 4, member e
2.552	229.681	Il1b	interleukin 1 beta
2.552	32.371	Atxn7l3b	ataxin 7-like 3B
2.551	42.228	Eps8l3	EPS8-like 3
2.549	28.069	Pak1	p21 protein (Cdc42/Rac)-activated kinase 1
2.548	26.348	Erg	avian erythroblastosis virus E-26 (v-ets) oncogene related
2.534	43.015	Furin	furin (paired basic amino acid cleaving enzyme)
2.534	168.924	Ifitm1	interferon induced transmembrane protein 1
2.527	253.776	Mefv	Mediterranean fever
2.526	19.465	Pgs1	phosphatidylglycerophosphate synthase 1
2.525	146.954	Trem1	triggering receptor expressed on myeloid cells 1
2.522	102.658	Hdac4	histone deacetylase 4
2.518	39.108	2010002M12Rik	RIKEN cDNA 2010002M12 gene
2.513	41.224	Rybp	RING1 and YY1 binding protein
2.510	86.943	Shisa5	shisa homolog 5 (Xenopus laevis)
2.507	53.596	Fam134b	family with sequence similarity 134, member B
2.507	92.038	Slc22a20	solute carrier family 22 (organic anion transporter), member 20
2.504	64.249	Hk3	hexokinase 3
2.497	139.982	Lmnb1	lamin B1
2.497	128.237	Gcnt1	glucosaminyl (N-acetyl) transferase 1, core 2
2.495	98.560	Cd300ld	CD300 molecule-like family member d
2.493	27.925	Notch2	notch 2
1.479	97.208	Sox2	SRY (sex determining region Y)-box 2

Supplementary Table 3: ***Unique H3K27ac peaks in ADC samples***

Unique somatic peaks associated with protein coding genes, top 185 and an additional 2 selected loci, of a possible 1820 with Log2FC>2.5, p value<1E-10

LOG2FC	Negative LOG10 (p value)	Gene Name	Gene Description
-7.845	51.798	Cobl	cordon-bleu WH2 repeat
-7.533	41.356	Tmem121	transmembrane protein 121
-7.260	33.913	Rdh14	retinol dehydrogenase 14 (all-trans and 9-cis)
-6.938	26.786	Ppp3cc	protein phosphatase 3, catalytic subunit, gamma isoform
-6.923	26.489	Hecw1	HECT, C2 and WW domain containing E3 ubiquitin protein ligase 1
-6.892	25.896	Gm8369	predicted gene 8369
-6.861	25.304	Tmem121	transmembrane protein 121
-6.675	22.049	1700071K01Rik	RIKEN cDNA 1700071K01 gene
-6.543	19.982	1110059E24Rik	RIKEN cDNA 1110059E24 gene
-6.523	19.687	Fut8	fucosyltransferase 8
-6.462	18.802	Crb2	crumbs homolog 2 (Drosophila)
-6.440	18.507	Kcnk1	potassium channel, subfamily K, member 1
-6.419	18.213	Phtf2	putative homeodomain transcription factor 2
-6.397	17.918	Gm8369	predicted gene 8369
-6.353	17.330	Fgfr1	fibroblast growth factor receptor 1
-6.330	17.036	Usp12	ubiquitin specific peptidase 12
-6.284	16.448	Socs1	suppressor of cytokine signaling 1
-6.211	15.567	Sall4	sal-like 4 (Drosophila)
-6.186	15.273	Grap2	GRB2-related adaptor protein 2
-6.160	14.980	Spry4	sprouty homolog 4 (Drosophila)

-6.108	14.394	Epha4	Eph receptor A4
-6.108	14.394	Ets1	E26 avian leukemia oncogene 1, 5' domain
-6.108	14.394	Tmem123	transmembrane protein 123
-6.081	14.101	Tmem121	transmembrane protein 121
-6.053	13.808	Plekhh2	pleckstrin homology domain containing, family H (with MyTH4 domain) member 2
-6.025	13.516	Luzp2	leucine zipper protein 2
-6.025	13.516	Sult5a1	sulfotransferase family 5A, member 1
-5.997	13.224	Cr2	complement receptor 2
-5.997	13.224	Usp15	ubiquitin specific peptidase 15
-5.997	13.224	Zfp703	zinc finger protein 703
-5.968	12.931	Gm5082	predicted gene 5082
-5.968	12.931	Sh2d2a	SH2 domain protein 2A
-5.968	12.931	Tsen2	tRNA splicing endonuclease 2 homolog (S. cerevisiae)
-5.947	30.246	Olfr164	olfactory receptor 164
-5.938	12.639	F2rl1	coagulation factor II (thrombin) receptor-like 1
-5.938	12.639	Isca2	iron-sulfur cluster assembly 2 homolog (S. cerevisiae)
-5.938	12.639	Lingo2	leucine rich repeat and Ig domain containing 2
-5.938	12.639	Npy	neuropeptide Y
-5.877	12.056	Palld	palladin, cytoskeletal associated protein
-5.877	12.056	Zfp354b	zinc finger protein 354B
-5.845	11.765	Anp32a	acidic (leucine-rich) nuclear phosphoprotein 32 family, member A
-5.845	11.765	Cystm1	cysteine-rich transmembrane module containing 1
-5.845	11.765	Osbpl6	oxysterol binding protein-like 6
-5.845	11.765	Slc2a7	solute carrier family 2 (facilitated glucose transporter), member 7
-5.845	11.765	Zswim6	zinc finger SWIM-type containing 6
-5.841	27.901	Nol11	nucleolar protein 11
-5.812	11.474	Ddx10	DEAD (Asp-Glu-Ala-Asp) box polypeptide 10
-5.812	11.474	Maf	avian musculoaponeurotic fibrosarcoma (v-maf) AS42 oncogene homolog
-5.779	11.183	Lyzl1	lysozyme-like 1
-5.779	11.183	Sorbs1	sorbin and SH3 domain containing 1
-5.779	11.183	Tex36	testis expressed 36
-5.772	81.638	Onecut2	one cut domain, family member 2
-5.745	10.892	Dad1	defender against cell death 1
-5.745	10.892	Etaa1	Ewing tumor-associated antigen 1
-5.745	10.892	Pde6a	phosphodiesterase 6A, cGMP-specific, rod, alpha
-5.745	10.892	Zfp618	zinc finger protein 618
-5.741	25.854	Onecut3	one cut domain, family member 3
-5.711	10.602	Abca5	ATP-binding cassette, sub-family A (ABC1), member 5
-5.711	10.602	Ift122	intraflagellar transport 122
-5.711	10.602	Tekt2	tektin 2
-5.697	24.978	Sh2d1b1	SH2 domain protein 1B1
-5.675	10.311	Bend5	BEN domain containing 5
-5.675	10.311	Spdef	SAM pointed domain containing ets transcription factor
-5.638	10.021	B3gnt7	UDP-GlcNAc:betaGal beta-1,3-N-acetylglucosaminyltransferase 7
-5.638	10.021	Gas2	growth arrest specific 2
-5.638	10.021	Ptx3	pentraxin related gene
-5.638	10.021	Slc19a3	solute carrier family 19, member 3
-5.599	38.484	Cryl1	crystallin, lambda 1
-5.552	53.161	Ptprn2	protein tyrosine phosphatase, receptor type, N polypeptide 2
-5.549	37.025	Haao	3-hydroxyanthranilate 3,4-dioxygenase
-5.549	37.025	Wfs1	Wolfram syndrome 1 homolog (human)
-5.536	22.064	Med13l	mediator complex subunit 13-like
-5.484	21.192	Otol1	otolin 1 homolog (zebrafish)
-5.431	20.322	Pla2r1	phospholipase A2 receptor 1
-5.421	33.528	Car8	carbonic anhydrase 8
-5.375	19.452	Gldc	glycine decarboxylase
-5.356	19.163	Tox3	TOX high mobility group box family member 3
-5.336	18.873	Mettl15	methyltransferase like 15
-5.316	30.912	Ntm	neurotrimin
-5.304	30.622	Col6a2	collagen, type VI, alpha 2
-5.267	55.150	Camk1g	calcium/calmodulin-dependent protein kinase I gamma
-5.234	53.705	Mcpt8	mast cell protease 8
-5.204	28.304	Lax1	lymphocyte transmembrane adaptor 1
-5.171	16.564	Ets1	E26 avian leukemia oncogene 1, 5' domain
-5.171	16.564	Nrsn1	neurensin 1
-5.157	50.531	Alpi	alkaline phosphatase, intestinal
-5.151	27.148	Gcnt4	glucosaminyl (N-acetyl) transferase 4, core 2 (beta-1,6-N-acetylglucosaminyltransferase)
-5.137	26.859	Matn1	matrilin 1, cartilage matrix protein
-5.127	15.989	4921539E11Rik	RIKEN cDNA 4921539E11 gene
-5.127	15.989	4930523C07Rik	RIKEN cDNA 4930523C07 gene
-5.127	15.989	Dennd5b	DENN/MADD domain containing 5B
-5.127	15.989	Golt1a	golgi transport 1 homolog A (S. cerevisiae)
-5.127	15.989	Ostf1	osteoclast stimulating factor 1
-5.127	15.989	Tprg	transformation related protein 63 regulated
-5.104	15.702	Lpp	LIM domain containing preferred translocation partner in lipoma
-5.081	15.415	Pax5	paired box 5
-5.057	15.128	Scml4	sex comb on midleg-like 4 (Drosophila)

-5.057	15.128	Snx29	sorting nexin 29
-5.034	14.841	Cd209d	CD209d antigen
-5.034	14.841	Tgfbr2	transforming growth factor, beta receptor II
-5.024	24.552	Mei4	meiosis-specific, MEI4 homolog (S. cerevisiae)
-5.009	24.265	Cdk5rap2	CDK5 regulatory subunit associated protein 2
-4.985	14.268	Plac9a	placenta specific 9a
-4.977	33.980	Snx10	sorting nexin 10
-4.960	13.983	Satb2	special AT-rich sequence binding protein 2
-4.960	13.983	St8sia3	ST8 alpha-N-acetyl-neuraminide alpha-2,8-sialyltransferase 3
-4.960	13.983	Tmem121	transmembrane protein 121
-4.960	13.983	Zfpm1	zinc finger protein, multitype 1
-4.949	23.115	Proser2	proline and serine rich 2
-4.938	53.629	Camk2n1	calcium/calmodulin-dependent protein kinase II inhibitor 1
-4.933	22.828	Xylt1	xylosyltransferase 1
-4.917	22.541	Acnat2	acyl-coenzyme A amino acid N-acyltransferase 2
-4.917	22.541	Fam83d	family with sequence similarity 83, member D
-4.908	13.412	Clvs2	clavesin 2
-4.908	13.412	Nrip1	nuclear receptor interacting protein 1
-4.908	13.412	Ralyl	RALY RNA binding protein-like
-4.908	13.412	Shh	sonic hedgehog
-4.901	22.254	Serpind1	serine (or cysteine) peptidase inhibitor, clade D, member 1
-4.900	31.965	R3hdml	R3H domain containing-like
-4.885	21.968	Smad2	SMAD family member 2
-4.872	40.203	Rph3a	rabphilin 3A
-4.856	50.189	Rasgrp1	RAS guanyl releasing protein 1
-4.852	21.395	Fam19a5	family with sequence similarity 19, member A5
-4.852	21.395	Mboat2	membrane bound O-acyltransferase domain containing 2
-4.846	39.347	Atp8b1	ATPase, class I, type 8B, member 1
-4.837	39.062	Ppie	peptidylprolyl isomerase E (cyclophilin E)
-4.836	21.109	Onecut3	one cut domain, family member 3
-4.827	12.557	Filip1l	filamin A interacting protein 1-like
-4.827	12.557	Mpg	N-methylpurine-DNA glycosylase
-4.217	12.633	Foxa2	forkhead box A2
-4.217	12.633	Foxo1	forkhead box O1

Supplementary Table 4: *Unique H3K4me3 peaks in ADC samples*

Unique somatic peaks associated with protein coding genes, top 185 and an additional 2 selected loci, of a possible 2506 with Log2FC>1.4, p value<1E-10

LOG2FC	Negative LOG10 (p value)	Gene Name	Gene Description
-6.340	31.535	Adck3	aarF domain containing kinase 3
-5.599	18.213	Slc12a7	solute carrier family 12, member 7
-5.520	54.038	Paqr5	progestin and adipoQ receptor family member V
-5.464	16.448	Tnfsf8	tumor necrosis factor (ligand) superfamily, member 8
-5.391	15.567	Klra17	killer cell lectin-like receptor, subfamily A, member 17
-5.261	14.101	Elavl4	ELAV (embryonic lethal, abnormal vision, Drosophila)-like 4 (Hu antigen D)
-5.261	14.101	Map3k7	mitogen-activated protein kinase kinase kinase 7
-5.057	12.056	Nfatc3	nuclear factor of activated T cells, cytoplasmic, calcineurin dependent 3
-4.961	22.254	Faim3	Fas apoptotic inhibitory molecule 3
-4.896	21.109	Mylk	myosin, light polypeptide kinase
-4.855	10.311	Pydc3	pyrin domain containing 3
-4.791	19.397	Sh2d1b1	SH2 domain protein 1B1
-4.773	19.112	Tank	TRAF family member-associated Nf-kappa B activator
-4.755	18.827	Sec16b	SEC16 homolog B (S. cerevisiae)
-4.703	58.728	Mcpt1	mast cell protease 1
-4.662	8.865	Cadm4	cell adhesion molecule 4
-4.659	17.408	Scara5	scavenger receptor class A, member 5 (putative)
-4.621	8.577	Nt5c1a	5'-nucleotidase, cytosolic IA
-4.619	16.842	Tmem54	transmembrane protein 54
-4.564	61.328	Rpia	ribose 5-phosphate isomerase A
-4.536	15.713	Ankrd1	ankyrin repeat domain 1 (cardiac muscle)
-4.536	15.713	Ubtd2	ubiquitin domain containing 2
-4.533	8.001	Agpat4	1-acylglycerol-3-phosphate O-acyltransferase 4 (lysophosphatidic acid acyltransferase, delta)
-4.470	14.869	Pax5	paired box 5
-4.424	14.308	Klra18	killer cell lectin-like receptor, subfamily A, member 18
-4.386	69.408	Myof	myoferlin
-4.380	76.874	Ctse	cathepsin E
-4.355	21.414	Msln	mesothelin
-4.289	26.916	Olfr164	olfactory receptor 164
-4.285	55.596	Ly86	lymphocyte antigen 86
-4.278	100.790	R3hdml	R3H domain containing-like

-4.277	12.633	Ptprg	protein tyrosine phosphatase, receptor type, G
-4.273	55.039	Gimap3	GTPase, IMAP family member 3
-4.253	32.724	Ldlrad4	low density lipoprotein receptor class A domain containing 4
-4.251	12.355	Anks4b	ankyrin repeat and sterile alpha motif domain containing 4B
-4.224	12.078	Mecom	MDS1 and EVI1 complex locus
-4.224	82.210	Thpo	thrombopoietin
-4.224	31.892	Slamf6	SLAM family member 6
-4.197	11.801	Ano1	anoctamin 1, calcium activated chloride channel
-4.197	11.801	Gpr132	G protein-coupled receptor 132
-4.185	38.132	Resp18	regulated endocrine-specific protein 18
-4.170	11.525	Cd2	CD2 antigen
-4.168	37.576	Gimap7	GTPase, IMAP family member 7
-4.151	18.044	Msx1	msh homeobox 1
-4.133	17.765	C530008M17Rik	RIKEN cDNA C530008M17 gene
-4.115	35.910	Cd19	CD19 antigen
-4.106	23.025	Onecut2	one cut domain, family member 2
-4.076	16.929	Ccr7	chemokine (C-C motif) receptor 7
-4.074	72.214	A430107P09Rik	RIKEN cDNA A430107P09 gene
-4.057	16.651	Gna14	guanine nucleotide binding protein, alpha 14
-4.017	16.096	Apitd1	apoptosis-inducing, TAF9-like domain 1
-4.013	32.869	Gpr18	G protein-coupled receptor 18
-3.997	15.819	Dnajc22	DnaJ (Hsp40) homolog, subfamily C, member 22
-3.993	9.877	Camk2n1	calcium/calmodulin-dependent protein kinase II inhibitor 1
-3.993	9.877	Commd8	COMM domain containing 8
-3.993	9.877	Ippk	inositol 1,3,4,5,6-pentakisphosphate 2-kinase
-3.993	9.877	S1pr1	sphingosine-1-phosphate receptor 1
-3.976	20.547	Sncb	synuclein, beta
-3.974	37.053	Prss2	protease, serine 2
-3.961	9.604	Itgae	integrin alpha E, epithelial-associated
-3.950	41.523	Mzb1	marginal zone B and B1 cell-specific protein 1
-3.935	14.990	Clu	clusterin
-3.934	24.746	Tff1	trefoil factor 1
-3.929	9.332	Atp8b1	ATPase, class I, type 8B, member 1
-3.929	9.332	Gpr133	G protein-coupled receptor 133
-3.896	9.061	Gprin1	G protein-regulated inducer of neurite outgrowth 1
-3.896	9.061	Lrrc66	leucine rich repeat containing 66
-3.873	109.291	Smpd3	sphingomyelin phosphodiesterase 3, neutral
-3.862	8.790	Galnt5	UDP-N-acetyl-alpha-D-galactosamine:polypeptide N-acetylgalactosaminyltransferase 5
-3.862	8.790	Rapgef6	Rap guanine nucleotide exchange factor (GEF) 6
-3.862	8.790	Ybx3	Y box protein 3
-3.847	13.890	Sned1	sushi, nidogen and EGF-like domains 1
-3.832	18.092	Rbfox1	RNA binding protein, fox-1 homolog (C. elegans) 1
-3.827	8.520	Thbs1	thrombospondin 1
-3.825	13.616	Gcm2	glial cells missing homolog 2 (Drosophila)
-3.792	21.769	Cd83	CD83 antigen
-3.764	154.940	Sall4	sal-like 4 (Drosophila)
-3.764	17.009	Ccr6	chemokine (C-C motif) receptor 6
-3.759	54.695	Cd2	CD2 antigen
-3.754	12.796	Ptp4a3	protein tyrosine phosphatase 4a3
-3.728	16.469	Ano1	anoctamin 1, calcium activated chloride channel
-3.722	20.427	Clstn3	calsyntenin 3
-3.722	20.427	Ttc30a1	tetratricopeptide repeat domain 30A1
-3.655	11.710	Gabrp	gamma-aminobutyric acid (GABA) A receptor, pi
-3.655	11.710	Gramd4	GRAM domain containing 4
-3.655	11.710	Slc38a8	solute carrier family 38, member 8
-3.629	11.439	Txndc5	thioredoxin domain containing 5
-3.618	18.562	Vmn2r11	vomeronasal 2, receptor 11
-3.609	30.473	Muc5b	mucin 5, subtype B, tracheobronchial
-3.609	42.916	Trpa1	transient receptor potential cation channel, subfamily A, member 1
-3.608	55.627	Cxcr5	chemokine (C-X-C motif) receptor 5
-3.602	11.170	Adcy7	adenylate cyclase 7
-3.602	11.170	Insc	inscuteable homolog (Drosophila)
-3.600	30.208	Kcnip1	Kv channel-interacting protein 1
-3.596	14.595	Abcc4	ATP-binding cassette, sub-family C (CFTR/MRP), member 4
-3.591	22.542	Ptpn14	protein tyrosine phosphatase, non-receptor type 14
-3.578	46.364	Ptprt	protein tyrosine phosphatase, receptor type, T
-3.575	10.901	Il2rb	interleukin 2 receptor, beta chain
-3.556	14.063	Il21r	interleukin 21 receptor
-3.542	28.619	Thpo	thrombopoietin
-3.539	17.241	Hira	histone cell cycle regulation defective homolog A (S. cerevisiae)
-3.535	13.798	Pbx1	pre B cell leukemia homeobox 1
-3.522	28.092	Olfr164	olfactory receptor 164
-3.520	10.364	Bai1	brain-specific angiogenesis inhibitor 1
-3.520	10.364	Rpia	ribose 5-phosphate isomerase A
-3.515	13.534	Onecut2	one cut domain, family member 2
-3.513	24.385	St8sia3	ST8 alpha-N-acetyl-neuraminide alpha-2,8-sialyltransferase 3
-3.491	10.097	Camk2d	calcium/calmodulin-dependent protein kinase II, delta

-3.467	48.740	Aff3	AF4/FMR2 family, member 3
-3.450	12.743	Cd247	CD247 antigen
-3.442	40.797	Spdef	SAM pointed domain containing ets transcription factor
-3.432	69.389	Lgals6	lectin, galactose binding, soluble 6
-3.432	9.564	Cd5	CD5 antigen
-3.432	9.564	Duxbl3	double homeobox B-like 3
-3.426	46.902	Ern2	endoplasmic reticulum (ER) to nucleus signalling 2
-3.406	49.305	Klra18	killer cell lectin-like receptor, subfamily A, member 18
-3.404	15.149	Nol11	nucleolar protein 11
-3.399	42.406	Capzb	capping protein (actin filament) muscle Z-line, beta
-3.399	42.406	Rgs10	regulator of G-protein signalling 10
-3.376	24.429	Spp2	secreted phosphoprotein 2
-3.371	9.035	Gm8369	predicted gene 8369
-3.371	9.035	Nrarp	Notch-regulated ankyrin repeat protein
-3.371	9.035	Sulf1	sulfatase 1
-3.371	9.035	Wwc1	WW, C2 and coiled-coil domain containing 1
-3.369	18.309	Nfasc	neurofascin
-3.360	57.542	Pld4	phospholipase D family, member 4
-3.355	20.977	Elfn2	leucine rich repeat and fibronectin type III, extracellular 2
-3.354	18.047	Cgrrf1	cell growth regulator with ring finger domain 1
-3.354	18.047	Sh2d1b2	SH2 domain protein 1B2
-3.352	40.578	Gm14461	predicted gene 14461
-3.349	14.372	Ankdd1b	ankyrin repeat and death domain containing 1B
-3.349	14.372	Trim44	tripartite motif-containing 44
-3.339	8.771	Ptprn	protein tyrosine phosphatase, receptor type, N
-3.337	11.437	Skor2	SKI family transcriptional corepressor 2
-3.328	5.353	Rnf19b	ring finger protein 19B
-3.326	52.529	Acan	aggrecan
-3.320	23.135	Cd79a	CD79A antigen (immunoglobulin-associated alpha)
-3.313	11.178	Eid1	EP300 interacting inhibitor of differentiation 1
-3.312	13.857	Trim58	tripartite motif-containing 58
-3.307	8.508	Bsg	basigin
-3.307	8.508	Cd22	CD22 antigen
-3.307	8.508	Postn	periostin, osteoblast specific factor
-3.305	35.825	Dock8	dedicator of cytokinesis 8
-3.302	19.940	Siglec1	sialic acid binding Ig-like lectin 1, sialoadhesin
-3.289	10.919	Stk32b	serine/threonine kinase 32B
-3.285	40.918	AI607873	expressed sequence AI607873
-3.278	95.042	Clu	clusterin
-3.274	8.246	Prss2	protease, serine 2
-3.260	16.487	Sla2	Src-like-adaptor 2
-3.258	52.102	Lhx8	LIM homeobox protein 8
-3.240	7.985	Adtrp	androgen dependent TFPI regulating protein
-3.240	7.985	Anp32a	acidic (leucine-rich) nuclear phosphoprotein 32 family, member A
-3.240	7.985	Sall4	sal-like 4 (Drosophila)
-3.239	10.404	Plet1	placenta expressed transcript 1
-3.239	10.404	Susd2	sushi domain containing 2
-3.220	18.396	Btbd11	BTB (POZ) domain containing 11
-3.213	12.580	Slc45a3	solute carrier family 45, member 3
-3.213	10.148	Akap13	A kinase (PRKA) anchor protein 13
-3.213	10.148	Efna5	ephrin A5
-3.213	10.148	Foxp1	forkhead box P1
-3.213	10.148	Tox	thymocyte selection-associated high mobility group box
-3.210	44.166	Kng2	kininogen 2
-3.205	7.725	Ikzf3	IKAROS family zinc finger 3
-2.372	140.221	Sftpb	surfactant associated protein B
-2.077	61.328	Foxa2	forkhead box A2

Supplementary Table 5: *ROSE algorithm on H3K27ac peaks in SCC samples*

top 200 somatic super-enhancers associated with protein coding genes, and microRNA 21a

Ranking	Gene Name	Gene Description
1	Tomm20	translocase of outer mitochondrial membrane 20 homolog (yeast)
2	Mir21a	microRNA 21a
3	Ergic1	endoplasmic reticulum-golgi intermediate compartment (ERGIC) 1
4	Mcl1	myeloid cell leukemia sequence 1
5	Ifitm3	interferon induced transmembrane protein 3
6	1600014C10Rik	RIKEN cDNA 1600014C10 gene
7	Zfp217	zinc finger protein 217
8	Socs3	suppressor of cytokine signaling 3
9	Bcl2l1	BCL2-like 1
10	Furin	furin (paired basic amino acid cleaving enzyme)
11	Myh9	myosin, heavy polypeptide 9, non-muscle
12	Ier5	immediate early response 5
13	E030018B13Rik	RIKEN cDNA E030018B13 gene

14	Pxn	paxillin
15	Irf2bp2	interferon regulatory factor 2 binding protein 2
16	Lrp1	low density lipoprotein receptor-related protein 1
17	Mbp	myelin basic protein
18	Ccdc12	coiled-coil domain containing 12
19	Ptpn1	protein tyrosine phosphatase, non-receptor type 1
20	Sik1	salt inducible kinase 1
21	Tatdn2	TatD DNase domain containing 2
22	Irf1	interferon regulatory factor 1
23	Rara	retinoic acid receptor, alpha
24	Sorl1	sortilin-related receptor, LDLR class A repeats-containing
25	Lyst	lysosomal trafficking regulator
26	Cap1	CAP, adenylate cyclase-associated protein 1 (yeast)
27	Gsr	glutathione reductase
28	Cd14	CD14 antigen
29	Spatc1	spermatogenesis and centriole associated 1
30	Rin3	Ras and Rab interactor 3
31	Ly6e	lymphocyte antigen 6 complex, locus E
32	Aff1	AF4/FMR2 family, member 1
33	Iqgap1	IQ motif containing GTPase activating protein 1
34	Incenp	inner centromere protein
35	Tgfbr1	transforming growth factor, beta receptor I
36	S100a9	S100 calcium binding protein A9 (calgranulin B)
37	Btg2	B cell translocation gene 2, anti-proliferative
38	Pfkfb3	6-phosphofructo-2-kinase/fructose-2,6-biphosphatase 3
39	Trim8	tripartite motif-containing 8
40	Fam49b	family with sequence similarity 49, member B
41	Tspan14	tetraspanin 14
42	Cxcr2	chemokine (C-X-C motif) receptor 2
43	Ptpn22	protein tyrosine phosphatase, non-receptor type 22 (lymphoid)
44	Zmiz1	zinc finger, MIZ-type containing 1
45	Irf2bpl	interferon regulatory factor 2 binding protein-like
46	Fgr	Gardner-Rasheed feline sarcoma viral (Fgr) oncogene homolog
47	Actb	actin, beta
48	Rassf6	Ras association (RalGDS/AF-6) domain family member 6
49	Midn	midnolin
50	Csf3r	colony stimulating factor 3 receptor (granulocyte)
51	Ndst1	N-deacetylase/N-sulfotransferase (heparan glucosaminyl) 1
52	Asprv1	aspartic peptidase, retroviral-like 1
53	Irf2bpl	interferon regulatory factor 2 binding protein-like
54	Bcl3	B cell leukemia/lymphoma 3
55	Junb	jun B proto-oncogene
56	Litaf	LPS-induced TN factor
57	Cflar	CASP8 and FADD-like apoptosis regulator
58	Osgin1	oxidative stress induced growth inhibitor 1
59	Clvs1	clavesin 1
60	Slfn2	schlafen 2
61	Fbxl5	F-box and leucine-rich repeat protein 5
62	Cmip	c-Maf inducing protein
63	Pstpip1	proline-serine-threonine phosphatase-interacting protein 1
64	Mknk2	MAP kinase-interacting serine/threonine kinase 2
65	Rassf3	Ras association (RalGDS/AF-6) domain family member 3
66	Eea1	early endosome antigen 1
67	Sgms1	sphingomyelin synthase 1
68	Actn1	actinin, alpha 1
69	Tgm2	transglutaminase 2, C polypeptide
70	Ubxn11	UBX domain protein 11
71	Hist1h3e	histone cluster 1, H3e
72	Fkbp8	FK506 binding protein 8
73	Ifnar2	interferon (alpha and beta) receptor 2
74	Il4ra	interleukin 4 receptor, alpha
75	Zfp608	zinc finger protein 608
76	Ninj2	ninjurin 2
77	Spi1	spleen focus forming virus (SFFV) proviral integration oncogene
78	Krt80	keratin 80
79	Il1r2	interleukin 1 receptor, type II
80	Vps54	vacuolar protein sorting 54 (yeast)
81	Cd44	CD44 antigen
82	Ptpn6	protein tyrosine phosphatase, non-receptor type 6
83	Adam19	a disintegrin and metallopeptidase domain 19 (meltrin beta)
84	Hic1	hypermethylated in cancer 1
85	Tnrc18	trinucleotide repeat containing 18
86	Atf1	activating transcription factor 1
87	Pim1	proviral integration site 1
88	Tbl1xr1	transducin (beta)-like 1X-linked receptor 1
89	Plekha2	pleckstrin homology domain-containing, family A (phosphoinositide binding specific) member 2
90	Ifngr1	interferon gamma receptor 1
91	Xbp1	X-box binding protein 1

92	Cxcl1	chemokine (C-X-C motif) ligand 1
93	S100a3	S100 calcium binding protein A3
94	Armc12	armadillo repeat containing 12
95	Hif1a	hypoxia inducible factor 1, alpha subunit
96	Lasp1	LIM and SH3 protein 1
97	Irf2bp2	interferon regulatory factor 2 binding protein 2
98	Pf4	platelet factor 4
99	Nek6	NIMA (never in mitosis gene a)-related expressed kinase 6
100	Zfp36	zinc finger protein 36
101	Cd9	CD9 antigen
102	Rab7	RAB7, member RAS oncogene family
103	Ndufs8	NADH dehydrogenase (ubiquinone) Fe-S protein 8
104	Gnai2	guanine nucleotide binding protein (G protein), alpha inhibiting 2
105	Cyth4	cytohesin 4
106	Rhof	ras homolog gene family, member f
107	Coro2a	coronin, actin binding protein 2A
108	Dnajb12	DnaJ (Hsp40) homolog, subfamily B, member 12
109	Fos	FBJ osteosarcoma oncogene
110	Itgb2	integrin beta 2
111	Msl1	male-specific lethal 1 homolog (Drosophila)
112	Snx29	sorting nexin 29
113	Notch1	notch 1
114	Rab31	RAB31, member RAS oncogene family
115	C1qtnf9	C1q and tumor necrosis factor related protein 9
116	Jun	jun proto-oncogene
117	Ctsd	cathepsin D
118	1110007C09Rik	RIKEN cDNA 1110007C09 gene
119	Fosl2	fos-like antigen 2
120	2810408A11Rik	RIKEN cDNA 2810408A11 gene
121	Wasf2	WAS protein family, member 2
122	Cdkn2aipnl	CDKN2A interacting protein N-terminal like
123	Inpp5d	inositol polyphosphate-5-phosphatase D
124	Phlda1	pleckstrin homology-like domain, family A, member 1
125	Cubn	cubilin (intrinsic factor-cobalamin receptor)
126	Alcam	activated leukocyte cell adhesion molecule
127	Tfeb	transcription factor EB
128	Tpm1	tropomyosin 1, alpha
129	Sh3bp2	SH3-domain binding protein 2
130	Tctex1d4	Tctex1 domain containing 4
131	C5ar2	complement component 5a receptor 2
132	Fcgr3	Fc receptor, IgG, low affinity III
133	Ly6e	lymphocyte antigen 6 complex, locus E
134	Bcl6	B cell leukemia/lymphoma 6
135	Ccdc80	coiled-coil domain containing 80
136	Cux1	cut-like homeobox 1
137	Cxcr4	chemokine (C-X-C motif) receptor 4
138	N4bp1	NEDD4 binding protein 1
139	Zfhx3	zinc finger homeobox 3
140	Lcp1	lymphocyte cytosolic protein 1
141	Tmem140	transmembrane protein 140
142	Kdm6b	KDM1 lysine (K)-specific demethylase 6B
143	Tgif1	TGFB-induced factor homeobox 1
144	Nfkbia	nuclear factor of kappa light polypeptide gene enhancer in B cells inhibitor, alpha
145	Cotl1	coactosin-like 1 (Dictyostelium)
146	Entpd1	ectonucleoside triphosphate diphosphohydrolase 1
147	Swap70	SWA-70 protein
148	Hnrnpl	heterogeneous nuclear ribonucleoprotein L
149	Rreb1	ras responsive element binding protein 1
150	Dhrs3	dehydrogenase/reductase (SDR family) member 3
151	Tbc1d14	TBC1 domain family, member 14
152	Ccdc91	coiled-coil domain containing 91
153	Sgms1	sphingomyelin synthase 1
154	Runx1	runt related transcription factor 1
155	Rbm47	RNA binding motif protein 47
156	Csf1	colony stimulating factor 1 (macrophage)
157	Prss22	protease, serine 22
158	Klhl6	kelch-like 6
159	Fpr1	formyl peptide receptor 1
160	Osbpl9	oxysterol binding protein-like 9
161	Akap13	A kinase (PRKA) anchor protein 13
162	Klf3	Kruppel-like factor 3 (basic)
163	Lipc	lipase, hepatic
164	Pla2g7	phospholipase A2, group VII (platelet-activating factor acetylhydrolase, plasma)
165	Chd2	chromodomain helicase DNA binding protein 2
166	Smad7	SMAD family member 7
167	Agps	alkylglycerone phosphate synthase
168	Cdt1	chromatin licensing and DNA replication factor 1
169	Pigx	phosphatidylinositol glycan anchor biosynthesis, class X

170	Amer2	APC membrane recruitment 2
171	Zbtb42	zinc finger and BTB domain containing 42
172	Fam129b	family with sequence similarity 129, member B
173	Egln3	egl-9 family hypoxia-inducible factor 3
174	Tle3	transducin-like enhancer of split 3, homolog of Drosophila E(spl)
175	Pacsin2	protein kinase C and casein kinase substrate in neurons 2
176	Tpst2	protein-tyrosine sulfotransferase 2
177	Ly6a	lymphocyte antigen 6 complex, locus A
178	Fli1	Friend leukemia integration 1
179	Ubc	ubiquitin C
180	Pde4b	phosphodiesterase 4B, cAMP specific
181	6430531B16Rik	RIKEN cDNA 6430531B16 gene
182	Klhdc8a	kelch domain containing 8A
183	Rin3	Ras and Rab interactor 3
184	Ier3	immediate early response 3
185	Zfp541	zinc finger protein 541
186	Ankrd11	ankyrin repeat domain 11
187	Tnfrsf26	tumor necrosis factor receptor superfamily, member 26
188	Erg	avian erythroblastosis virus E-26 (v-ets) oncogene related
189	Vps37b	vacuolar protein sorting 37B (yeast)
190	Ptma	prothymosin alpha
191	Lrrfip1	leucine rich repeat (in FLII) interacting protein 1
192	Tnfrsf1b	tumor necrosis factor receptor superfamily, member 1b
193	Zfp608	zinc finger protein 608
194	Zfp503	zinc finger protein 503
195	Rhob	ras homolog gene family, member B
196	Slc16a11	solute carrier family 16 (monocarboxylic acid transporters), member 11
197	Dusp6	dual specificity phosphatase 6
198	Dhrs3	dehydrogenase/reductase (SDR family) member 3
199	Tnfaip2	tumor necrosis factor, alpha-induced protein 2
200	Pagr1a	PAXIP1 associated glutamate rich protein 1A

Supplementary Table 6: *ROSE algorithm on H3K27ac peaks in ADC samples*

top 200 somatic super-enhancers associated with protein coding genes, and microRNA 21a

Ranking	Gene Name	Gene Description
1	Irf2bp2	interferon regulatory factor 2 binding protein 2
2	Mir21a	microRNA 21a
3	Sik1	salt inducible kinase 1
4	Rad51b	RAD51 homolog B
5	Myh9	myosin, heavy polypeptide 9, non-muscle
6	Ddit4	DNA-damage-inducible transcript 4
7	Mcl1	myeloid cell leukemia sequence 1
8	Ergic1	endoplasmic reticulum-golgi intermediate compartment (ERGIC) 1
9	Socs3	suppressor of cytokine signaling 3
10	Abhd2	abhydrolase domain containing 2
11	Ly6e	lymphocyte antigen 6 complex, locus E
12	Sgms1	sphingomyelin synthase 1
13	Dhrs3	dehydrogenase/reductase (SDR family) member 3
14	Zmiz1	zinc finger, MIZ-type containing 1
15	Carns1	carnosine synthase 1
16	Bcl2l1	BCL2-like 1
17	Trim8	tripartite motif-containing 8
18	Smad3	SMAD family member 3
19	Cblc	Casitas B-lineage lymphoma c
20	Irf2	interferon regulatory factor 2
21	Capzb	capping protein (actin filament) muscle Z-line, beta
22	Irf2bpl	interferon regulatory factor 2 binding protein-like
23	E030018B13Rik	RIKEN cDNA E030018B13 gene
24	Lta	lymphotoxin A
25	Sdc1	syndecan 1
26	Ski	ski sarcoma viral oncogene homolog (avian)
27	Hyal3	hyaluronoglucosaminidase 3
28	Dusp7	dual specificity phosphatase 7
29	Il4ra	interleukin 4 receptor, alpha
30	Zfp217	zinc finger protein 217
31	Htra4	HtrA serine peptidase 4
32	Pmepa1	prostate transmembrane protein, androgen induced 1
33	Kxd1	KxDL motif containing 1
34	Tpm1	tropomyosin 1, alpha
35	Cebpa	CCAAT/enhancer binding protein (C/EBP), alpha
36	Pgpep1	pyroglutamyl-peptidase I

37	Actn1	actinin, alpha 1
38	St3gal1	ST3 beta-galactoside alpha-2,3-sialyltransferase 1
39	Plekhf1	pleckstrin homology domain containing, family F (with FYVE domain) member 1
40	Synpo	synaptopodin
41	Phlda1	pleckstrin homology-like domain, family A, member 1
42	Ier5	immediate early response 5
43	Sema4b	sema domain, immunoglobulin domain (Ig), transmembrane domain (TM) and short cytoplasmic domain, (semaphorin) 4B
44	Zmynd8	zinc finger, MYND-type containing 8
45	Crebzf	CREB/ATF bZIP transcription factor
46	Smad7	SMAD family member 7
47	Ndst1	N-deacetylase/N-sulfotransferase (heparan glucosaminyl) 1
48	Tspan14	tetraspanin 14
49	Slc34a3	solute carrier family 34 (sodium phosphate), member 3
50	Furin	furin (paired basic amino acid cleaving enzyme)
51	Btg2	B cell translocation gene 2, anti-proliferative
52	Ccdc12	coiled-coil domain containing 12
53	Fam129b	family with sequence similarity 129, member B
54	Rgs3	regulator of G-protein signaling 3
55	Plekhg2	pleckstrin homology domain containing, family G (with RhoGef domain) member 2
56	Tnfrsf1b	tumor necrosis factor receptor superfamily, member 1b
57	Adssl1	adenylosuccinate synthetase like 1
58	Ctsd	cathepsin D
59	Shb	src homology 2 domain-containing transforming protein B
60	Pxn	paxillin
61	Syngr2	synaptogyrin 2
62	Wasf2	WAS protein family, member 2
63	Sftpb	surfactant associated protein B
64	Col4a2	collagen, type IV, alpha 2
65	1600014C23Rik	RIKEN cDNA 1600014C23 gene
66	Elk3	ELK3, member of ETS oncogene family
67	Hif1a	hypoxia inducible factor 1, alpha subunit
68	Vgll4	vestigial like 4 (Drosophila)
69	Sipa1l3	signal-induced proliferation-associated 1 like 3
70	Lipc	lipase, hepatic
71	Tgif1	TGFB-induced factor homeobox 1
72	Tnrc18	trinucleotide repeat containing 18
73	Ak2	adenylate kinase 2
74	Rac2	RAS-related C3 botulinum substrate 2
75	Ech1	enoyl coenzyme A hydratase 1, peroxisomal
76	Myo1e	myosin IE
77	Ptpn1	protein tyrosine phosphatase, non-receptor type 1
78	Coro2a	coronin, actin binding protein 2A
79	Tmem107	transmembrane protein 107
80	Bcar3	breast cancer anti-estrogen resistance 3
81	Zfp36l1	zinc finger protein 36, C3H type-like 1
82	Pim1	proviral integration site 1
83	Junb	jun B proto-oncogene
84	Src	Rous sarcoma oncogene
85	Actb	actin, beta
86	B3gnt7	UDP-GlcNAc:betaGal beta-1,3-N-acetylglucosaminyltransferase 7
87	Rara	retinoic acid receptor, alpha
88	Tatdn2	TatD DNase domain containing 2
89	Fth1	ferritin heavy chain 1
90	Baiap2	brain-specific angiogenesis inhibitor 1-associated protein 2
91	Epha2	Eph receptor A2
92	Iqgap1	IQ motif containing GTPase activating protein 1
93	Krt8	keratin 8
94	Midn	midnolin
95	Pwwp2b	PWWP domain containing 2B
96	Mex3a	mex3 homolog A (C. elegans)
97	Sbno2	strawberry notch homolog 2 (Drosophila)
98	Ccr7	chemokine (C-C motif) receptor 7
99	S100a4	S100 calcium binding protein A4
100	Laptm4a	lysosomal-associated protein transmembrane 4A
101	Fcgr2b	Fc receptor, IgG, low affinity IIb
102	Foxp1	forkhead box P1
103	Fos	FBJ osteosarcoma oncogene
104	Cerk	ceramide kinase
105	Setd1b	SET domain containing 1B
106	Asprv1	aspartic peptidase, retroviral-like 1
107	Myof	myoferlin
108	Mknk2	MAP kinase-interacting serine/threonine kinase 2
109	Actn4	actinin alpha 4
110	Rassf2	Ras association (RalGDS/AF-6) domain family member 2
111	Ninj2	ninjurin 2

112	Ddx47	DEAD (Asp-Glu-Ala-Asp) box polypeptide 47
113	Hes1	hairy and enhancer of split 1 (Drosophila)
114	D8Ertd82e	DNA segment, Chr 8, ERATO Doi 82, expressed
115	Pdlim2	PDZ and LIM domain 2
116	Nr1d1	nuclear receptor subfamily 1, group D, member 1
117	Rreb1	ras responsive element binding protein 1
118	Sep9	septin 9
119	Lrp1	low density lipoprotein receptor-related protein 1
120	Fam49b	family with sequence similarity 49, member B
121	Pacsin2	protein kinase C and casein kinase substrate in neurons 2
122	Cdkn2b	cyclin-dependent kinase inhibitor 2B (p15, inhibits CDK4)
123	Mbp	myelin basic protein
124	Cyth1	cytohesin 1
125	Hspa8	heat shock protein 8
126	Itpk1	inositol 1,3,4-triphosphate 5/6 kinase
127	Ly86	lymphocyte antigen 86
128	Scgb1a1	secretoglobin, family 1A, member 1 (uteroglobin)
129	Mpv17l2	MPV17 mitochondrial membrane protein-like 2
130	Ypel3	yippee-like 3 (Drosophila)
131	Mpeg1	macrophage expressed gene 1
132	Dusp6	dual specificity phosphatase 6
133	Notch2	notch 2
134	Plau	plasminogen activator, urokinase
135	Irf2bpl	interferon regulatory factor 2 binding protein-like
136	Lama5	laminin, alpha 5
137	Piezo1	piezo-type mechanosensitive ion channel component 1
138	Fam53b	family with sequence similarity 53, member B
139	Ttc7	tetratricopeptide repeat domain 7
140	E230025N22Rik	Riken cDNA E230025N22 gene
141	Lrrfip1	leucine rich repeat (in FLII) interacting protein 1
142	Hnrnpa3	heterogeneous nuclear ribonucleoprotein A3
143	Gsr	glutathione reductase
144	Ldlrap1	low density lipoprotein receptor adaptor protein 1
145	Ppard	peroxisome proliferator activator receptor delta
146	S1pr2	sphingosine-1-phosphate receptor 2
147	Nek6	NIMA (never in mitosis gene a)-related expressed kinase 6
148	Armc12	armadillo repeat containing 12
149	Hfe	hemochromatosis
150	Adamts4	a disintegrin-like and metallopeptidase (reprolysin type) with thrombospondin type 1 motif, 4
151	Elmsan1	ELM2 and Myb/SANT-like domain containing 1
152	Ctsc	cathepsin C
153	Spag7	sperm associated antigen 7
154	Gpr146	G protein-coupled receptor 146
155	Lasp1	LIM and SH3 protein 1
156	Clic1	chloride intracellular channel 1
157	Gltscr1	glioma tumor suppressor candidate region gene 1
158	Aff1	AF4/FMR2 family, member 1
159	Rbm47	RNA binding motif protein 47
160	Pnrc2	proline-rich nuclear receptor coactivator 2
161	Zc3h4	zinc finger CCCH-type containing 4
162	Dok1	docking protein 1
163	Hpcal1	hippocalcin-like 1
164	Rassf3	Ras association (RalGDS/AF-6) domain family member 3
165	Bcl9l	B cell CLL/lymphoma 9-like
166	Spns2	spinster homolog 2
167	Myzap	myocardial zonula adherens protein
168	Ier5l	immediate early response 5-like
169	Pkig	protein kinase inhibitor, gamma
170	Vamp5	vesicle-associated membrane protein 5
171	Scd2	stearoyl-Coenzyme A desaturase 2
172	C1qtnf9	C1q and tumor necrosis factor related protein 9
173	Plac8	placenta-specific 8
174	Litaf	LPS-induced TN factor
175	Ifi47	interferon gamma inducible protein 47
176	Ccdc19	coiled-coil domain containing 19
177	Snai1	snail family zinc finger 1
178	Tgfbr2	transforming growth factor, beta receptor II
179	Nfkbia	nuclear factor of kappa light polypeptide gene enhancer in B cells inhibitor, alpha
180	Mgat5	mannoside acetylglucosaminyltransferase 5
181	Cflar	CASP8 and FADD-like apoptosis regulator
182	Cmip	c-Maf inducing protein
183	Polr2e	polymerase (RNA) II (DNA directed) polypeptide E
184	Rasa3	RAS p21 protein activator 3
185	Tgm2	transglutaminase 2, C polypeptide
186	BC051665	cDNA sequence BC051665
187	Synj2	synaptojanin 2

188	Pnpla2	patatin-like phospholipase domain containing 2
189	Metrnl	meteorin, glial cell differentiation regulator-like
190	Sdc4	syndecan 4
191	Igfbp7	insulin-like growth factor binding protein 7
192	Mrpl52	mitochondrial ribosomal protein L52
193	Bahcc1	BAH domain and coiled-coil containing 1
194	St6gal1	beta galactoside alpha 2,6 sialyltransferase 1
195	Slc9a3r1	solute carrier family 9 (sodium/hydrogen exchanger), member 3 regulator 1
196	Elmo3	engulfment and cell motility 3
197	Herpud1	homocysteine-inducible, endoplasmic reticulum stress-inducible, ubiquitin-like domain member 1
198	Adam19	a disintegrin and metallopeptidase domain 19 (meltrin beta)
199	Dyrk3	dual-specificity tyrosine-(Y)-phosphorylation regulated kinase 3
200	Sh3bp4	SH3-domain binding protein 4
